# Diagnostic challenges in postoperative pelvic infections associated with *Metamycoplasma hominis*: a two-case analysis using metagenomic sequencing

**DOI:** 10.3389/fcimb.2026.1823299

**Published:** 2026-04-29

**Authors:** Yusuke Takahashi, Ryuichi Minoda Sada, Hiroo Matsuo, Shungo Yamamoto, Shinya Matsuzaki, Aiko Okada, Atsuko Sunada, Miyuki Takao, Go Yamamoto, Ching-Kai Chuang, Chun-Hao Liu, Satoshi Kutsuna

**Affiliations:** 1 Department of Infection Control and Prevention, Graduate School of Medicine, The University of Osaka, Osaka, Japan; 2 Department of Transformative Analysis for Human Specimen, Graduate School of Medicine, The University of Osaka, Osaka, Japan; 3 Centre for Infectious Disease Education and Research, The University of Osaka, Osaka, Japan; 4 Department of General Internal Medicine, Tenri Hospital, Nara, Japan; 5 Department of Transformative Protection to Infectious Disease, Graduate School of Medicine, The University of Osaka, Osaka, Japan; 6 Department of Obstetrics and Gynecology, Graduate School of Medicine, The University of Osaka, Osaka, Japan; 7 Department of Clinical Laboratory, Graduate School of Medicine, The University of Osaka, Osaka, Japan; 8 Asia Pathogenomics Co., Ltd., Taipei, Taiwan; 9 Asia Pathogenomics Japan Co., Ltd., Tokyo, Japan

**Keywords:** 16S rRNA gene PCR, genital mollicutes, metagenomic next-generation sequencing, metamycoplasma hominis, postoperative pelvic infection

## Abstract

Postoperative gynecological infections may present diagnostic challenges, particularly in the presence of fastidious genital mollicutes and inherently mixed microbial DNA, both of which limit the diagnostic performance of microbiological methods, including Gram staining, conventional culture, 16S rRNA gene PCR followed by Sanger sequencing. This study aimed to illustrate the limitations of conventional microbiological methods in the diagnosis of gynecologic pelvic infections and highlight key considerations for the clinical use of metagenomic next-generation sequencing (mNGS), based on two contrasting cases of postoperative pelvic infections associated with *Metamycoplasma hominis* (*M. hominis*). In both cases, neither conventional culture nor 16S rRNA gene PCR/Sanger sequencing identified the causative organism, and shotgun mNGS was subsequently performed. Although the mNGS findings differed markedly between the two cases, *M. hominis* was considered the most plausible pathogen. These two cases show that the clinical relevance of organisms detected by mNGS should not be judged by read counts alone, particularly in non-sterile specimens or after antibiotic exposure. Even low-abundance reads may represent clinically meaningful pathogens when interpreted within the clinical context. They also highlighted the value of mNGS as a complementary diagnostic tool for gynecological pelvic infections when conventional diagnostic methods are intrinsically limited.

## 1 Introduction

Gynecological pelvic infections, including postoperative and postpartum cases, can be challenging to diagnose because fastidious genital mollicutes, such as *Mycoplasma* and *Ureaplasma* species, may be involved (Chikamatsu et al., 2024; Yagi et al., 2024). Accurate detection of these organisms is often difficult in clinical settings because they are invisible on Gram staining and grow slowly, forming only tiny colonies (Taylor-Robinson, 1996; Yeoh et al., 2023), which can lead to delayed initiation of appropriate therapy (Reissier et al., 2016; Ali et al., 2021).

Under these circumstances, genomic methods may have additional diagnostic value. 16S rRNA gene PCR followed by Sanger sequencing is widely used as a genomic diagnostic tool in clinical microbiology laboratories (Rampini et al., 2011; Fida et al., 2021); however, their utility is limited to specimens with mixed bacterial DNA (Winand et al., 2019; Zhang et al., 2019), a characteristic often observed in gynecological infections (Nogami et al., 2023). Metagenomic next-generation sequencing (mNGS) has emerged as a useful complementary diagnostic approach when conventional methods are limited (Gu et al., 2019). However, the appropriate clinical application of mNGS remains challenging, as standardized criteria for the interpretation of results have not yet been established (Chiu and Miller, 2019).

In this study, mNGS was applied to two cases of gynecological pelvic infection associated with *Metamycoplasma hominis* (formerly *Mycoplasma hominis)*, where conventional diagnostic methods failed to identify the causative pathogen. These two cases differed in specimen characteristics and clinical settings, which were reflected in their distinct mNGS profiles. This study provides practical insights into the interpretation of mNGS findings through a comparative analysis of two cases with distinct sequencing profiles, emphasizing the importance of integrating metagenomic data with clinical and specimen context.

## 2 Methods

### 2.1 Clinical specimens and conventional microbiology

Clinical specimens were obtained as part of routine diagnostic procedures. In Case 1, aspirated fluid from a postoperative pelvic abscess was collected under computed tomography guidance. In Case 2, a small volume of purulent intrauterine fluid was obtained transcervically using a Nelaton catheter under aseptic conditions. All specimens were subjected to Gram staining and routine aerobic and anaerobic cultures according to standard laboratory protocols. A portion of each specimen was subjected to molecular analyses, including 16S rRNA gene PCR/Sanger sequencing and mNGS.

### 2.2 DNA extraction

DNA was extracted from the clinical specimens immediately after sample collection using a commercially available nucleic acid extraction kit (MORA-EXTRACT; Advanced Microorganism Research Co., Ltd., Gifu, Japan). The extracted DNA was used for both 16S rRNA gene PCR/Sanger sequencing and mNGS analyses; detailed methods are provided in the **Supplementary Methods**.

### 2.3 16S rRNA gene PCR and Sanger sequencing

Broad-range bacterial 16S rRNA gene PCR was performed using the extracted DNA and standard universal primers 27F (5′-AGAGTTTGATCMTGGCTCAG-3′) and 1492R (5′-TACGGYTACCTTGTTCGACTT-3′) targeting approximately the V1–V9 regions. The PCR was performed immediately after DNA extraction in both cases. PCR products were subjected to Sanger sequencing using a core facility sequencing service at Osaka University following standard protocols.

### 2.4 Metagenomic next-generation sequencing

Shotgun mNGS was performed using the extracted DNA to identify potential causative pathogens. The timing of mNGS differed between the two cases: in Case 1, mNGS was performed as part of the diagnostic evaluation during the acute clinical course, whereas in Case 2, mNGS was performed retrospectively using extracted DNA that had been stored at 4 °C in RNase-free water provided with the extraction kit for approximately one year. Library preparation, sequencing, and bioinformatic analyses were conducted using a commercially available clinical mNGS pipeline (Asia Pathogenomics Co., Ltd., Taipei, Taiwan). Sequencing was performed using the Element Biosciences AVITI platform. After quality filtering, the host-derived reads were removed by alignment with the telomere-to-telomere human reference genome (T2T-CHM13). The remaining reads were aligned to a curated microbial reference database comprising bacterial, viral, fungal, and parasitic genomes. A comprehensive description of the mNGS workflow, including detailed library preparation protocols, is provided in the **Supplementary Methods**.

### 2.5 Quality control and read classification

Sequencing quality was assessed using standard metrics provided by the clinical mNGS pipeline, including read length and base quality scores. Low-quality reads and potential artifacts were removed before downstream analysis, and only reads that passed the quality control thresholds were used for microbial classification. Negative and process controls were included to evaluate the background signals and analytical specificity. (**Supplementary Tables 2**, **3**). A comprehensive description of bioinformatic parameters is provided in the **Supplementary Methods**.

### 2.6 Ethics approval

This study was reviewed and approved by the Ethics Committee of Osaka University (Approval No. 25422). Because clinical specimens were obtained as part of routine diagnostic and therapeutic procedures and all data were anonymized prior to analysis, written informed consent was not required. An opt-out option was provided to patients in accordance with institutional policies.

## Case description

### Case 1

A 26-year-old woman with no history of pregnancy was admitted for planned radical trachelectomy for cervical adenosquamous carcinoma (pT1b1 N0 M0, FIGO 2018 Stage IB1). On postoperative day (POD) 9, high fever and lower abdominal tenderness were observed. Contrast-enhanced computed tomography (CT) demonstrated fluid collection at the uterine anastomotic site and bilateral pelvic sidewalls with partial intralesional gas, consistent with a deep abscess. Piperacillin–tazobactam (18 g/day, administered in four divided doses) was initiated after obtaining blood and urine cultures. However, there was no clinical improvement, and both cultures remained negative.

**Figure 1:**
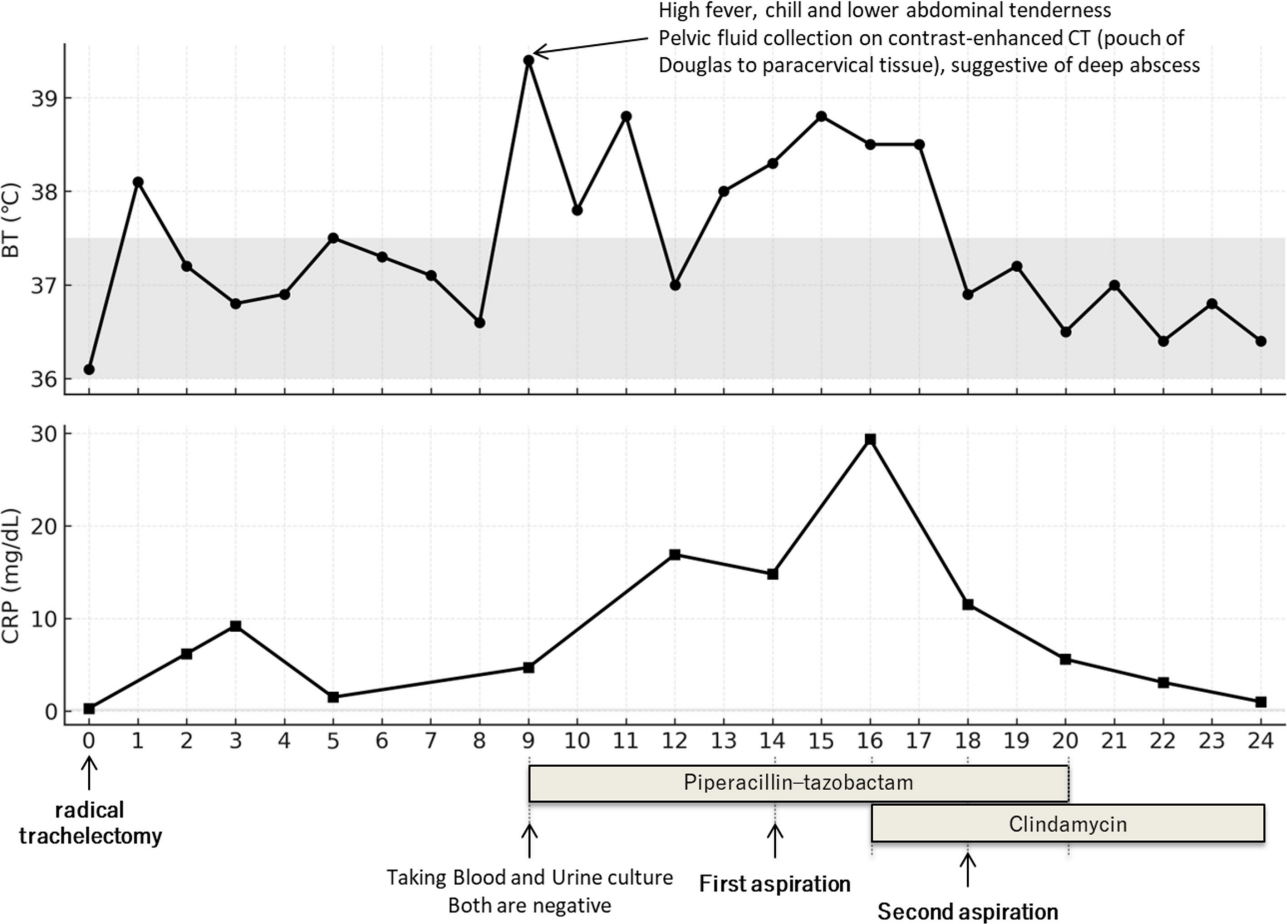
Clinical course of Case 1.

On POD 14, CT-guided percutaneous drainage of the collected fluid was performed, and the aspirated sample was subjected to microbiological testing. Gram staining revealed numerous leukocytes, but no organisms. Specimens were cultured on blood agar under aerobic and anaerobic conditions. No bacterial growth was observed after 72 h of incubation, which is the routine incubation period used in our laboratory. On POD 16, contrast-enhanced CT was performed again due to worsening clinical symptoms and laboratory data. The scan revealed a new pelvic fluid collection in the left iliac fossa, whereas the previous collection at the drainage site had decreased. Re-laparotomy with intra-abdominal irrigation and drainage was planned if her condition deteriorated further. Given the possibility of atypical pathogens not covered by β-lactams, clindamycin (2,700 mg/day, administered in three divided doses) was empirically added to piperacillin–tazobactam before obtaining the additional cultures. After the initiation of clindamycin treatment, the fever and inflammatory markers improved rapidly (**Figure 1**).

To identify the causative pathogen, a second aspiration was performed on POD 18. The specimens were subjected to culture, 16S rRNA gene PCR/Sanger sequencing, and shotgun mNGS. PCR of the 16S rRNA gene yielded a clear amplification band; however, Sanger sequencing did not reveal a specific organism because the chromatogram was uninterpretable (**Supplementary Figure 1**). The mNGS analysis revealed that *M. hominis* was the predominant organism, followed by *Ureaplasma parvum*. Detailed sequencing findings are described in the mNGS findings section. Given the favorable clinical response to clindamycin, *M. hominis* was considered the most likely primary pathogen, and no additional antimicrobial therapy was initiated to cover *U. parvum*. The aspirate was subjected to prolonged culture to facilitate isolation of *M. hominis*, as suggested by the mNGS results. After 9 days of incubation under anaerobic conditions on a blood agar plate, only two tiny colonies grew, which were identified as *M. hominis* (score value, 2.09) by MALDI-TOF MS system (MALDI Biotyper microflex LT/SH, Bruker Daltonics, Bremen, Germany) with MBT Compass software (version 4.1.90). However, the growth was insufficient for antimicrobial susceptibility testing. Based on these findings, *M. hominis* was diagnosed as the causative agent of the postoperative pelvic abscesses. Piperacillin–tazobactam treatment was switched to clindamycin monotherapy. The patient remained afebrile and was discharged 24 days postoperatively. At discharge, clindamycin was switched from intravenous to oral administration at an equivalent daily dose (2,700 mg). The total clindamycin treatment duration was 14 days. The patient has since been followed up without relapse.

### Case 2

A 31-year-old woman was admitted for labor induction at 41 weeks of gestation. During hospitalization, she developed an acute fever and was diagnosed with chorioamnionitis. Empiric antimicrobial therapy with ampicillin, clindamycin, and gentamicin was initiated without obtaining cultures, and an emergency cesarean section was performed on the same day. The surgery was completed successfully without any intraoperative complications; however, the fever worsened. On POD 2, the antimicrobial regimen was changed to meropenem monotherapy after blood cultures were obtained. The fever resolved promptly, and *Prevotella bivia* was subsequently identified in both blood cultures. The patient was initially treated with meropenem for chorioamnionitis complicated by *P. bivia* bacteremia. However, on POD 6, recurrent high fever with chills and new-onset marked lower abdominal pain were noted. Gynecological examination revealed uterine tenderness consistent with endometritis. Contrast-enhanced CT revealed an enlarged (subinvoluted) uterus compatible with endometritis with no evidence of extrauterine abscess or ovarian vein thrombosis. Transvaginal ultrasonography revealed a limited intrauterine fluid collection.

Based on these findings, the patient was diagnosed with postpartum endometritis. Although meropenem provides broad coverage, cell wall-deficient genital mollicutes (e.g., *M. hominis*) are not targeted by beta-lactams and were therefore considered in the differential diagnosis.

**Figure 2:**
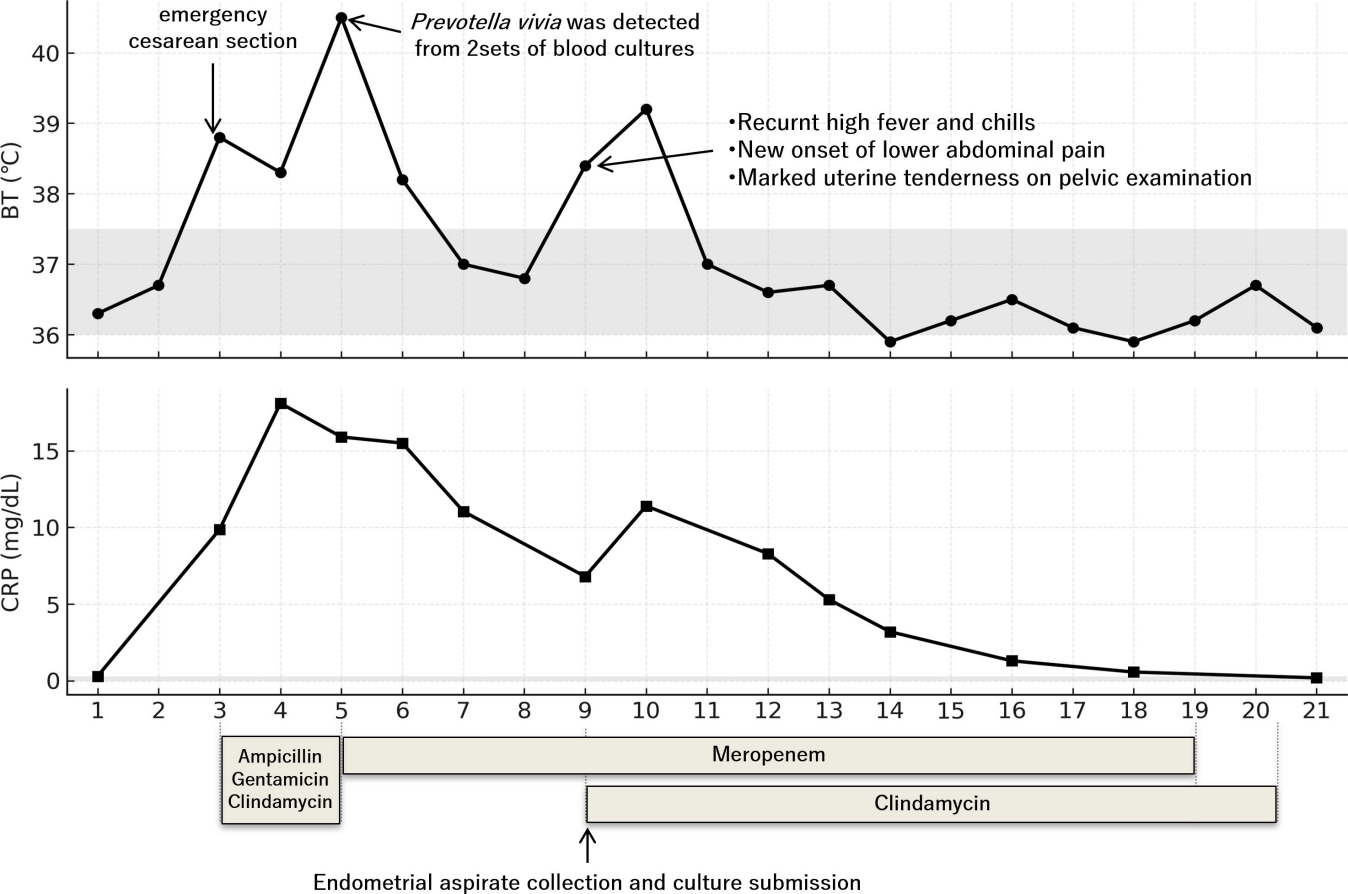
Clinical course of Case 2. The endometrial aspirate was obtained on postoperative day (POD) 6 before the reintroduction of clindamycin, which was initiated immediately after specimen collection. The patient had also received clindamycin several days earlier as part of the initial empiric treatment for chorioamnionitis.

The gynecology team noted that if endometritis worsened, hysterectomy might have become unavoidable. To identify the causative pathogen, a 12-Fr Nelaton catheter was inserted transcervically into the uterine cavity under aseptic conditions, and a small volume of purulent, blood-tinged intrauterine fluid (endometrial aspirate) was obtained. Gram staining revealed numerous leukocytes but no organisms. Considering the possible pathogens not sensitive to meropenem and not visible by Gram staining, including *M. hominis*, clindamycin (1,800 mg/day, administered in three divided doses) was added to the regimen immediately after collection of the endometrial aspirate on the same day. The specimens were also subjected to culture and 16S rRNA gene PCR, followed by Sanger sequencing. Culture was performed by inoculating the specimen onto blood agar plates under both aerobic and anaerobic conditions, as well as in a liquid medium (*Urea–Arginine LYO2*, Sysmex bioMérieux Co., Ltd., Tokyo, Japan). After the initiation of clindamycin treatment, fever and abdominal pain improved promptly (**Figure 2**). Meropenem was continued for 14 days as treatment for *P. bivia* bacteremia, and the total duration of clindamycin therapy was 14 days as treatment for endometritis. The patient was discharged and remained relapse-free during follow-up. Based on the favorable clinical response to clindamycin, an association with *M. hominis* was strongly suspected; however, no bacterial growth was observed in any of the cultures, even after prolonged incubation. PCR of the 16S rRNA gene yielded a clear amplification band; however, Sanger sequencing did not reveal a specific organism as the chromatogram was uninterpretable due to overlapping peaks, similar to Case 1. Therefore, the causative pathogen could not be identified. The extracted DNA was therefore stored at 4 °C in the laboratory. One year later, retrospective mNGS analysis of the stored samples was performed to elucidate the etiology. Detailed sequencing findings are described in the mNGS findings section. The analysis identified multiple bacterial reads with a high abundance of *P. bivia*, consistent with the blood culture results. Notably, 54 reads of *M. hominis* were detected despite prolonged storage of the specimen.

## 3 Results

### 3.1 mNGS findings in case 1

**Figure 3:**
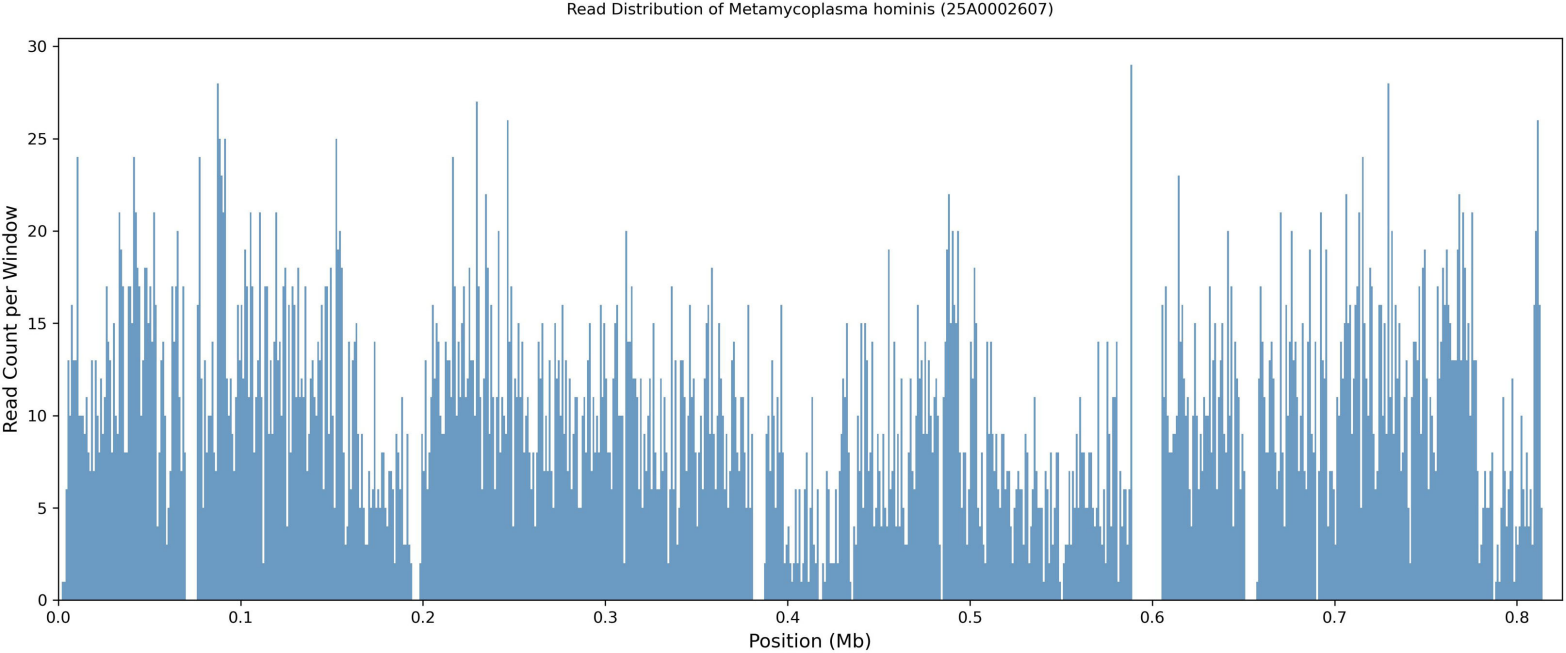
Read distribution of *M. hominis* detected by mNGS. The x-axis represents the genomic position of the *M. hominis* reference genome (GenBank accession: GCA_009664325.1), and the y-axis indicates the number of mapped reads per 1,000 bp window. A total of 7,917 reads were aligned to the reference genome. Reads were broadly distributed across the genome without regional bias.

DNA extracted from the pelvic abscess aspirate collected on POD 18 underwent shotgun mNGS. The following organisms were detected: *Metamycoplasma hominis*: 7,917 reads; *Ureaplasma parvum*: 905 reads; and *Lactobacillus iners*: 5 reads. *M. hominis* accounted for approximately 89% of all bacterial reads, with a genome coverage of 47% relative to the *M. hominis* reference genome (GenBank: GCA_009664325.1). Genome-wide read mapping demonstrated unbiased distribution across the reference genome (**Figure 3**; **Supplementary Table 1**).

### 3.2 mNGS findings in case 2

The DNA extracted from the endometrial aspirate collected on POD 6, which had been stored at 4 °C for one year, underwent shotgun metagenomic sequencing. The following organisms were detected: *Prevotella bivia*: 163,983 reads, *Ureaplasma parvum*: 10,029 reads, *Corynebacterium striatum*: 559 reads*, and Metamycoplasma hominis*: 54 reads. The *M. hominis* reads corresponded to a genome coverage of 0.18% relative to the reference genome (GenBank: GCA_009664325.1).

**Figure 4:**
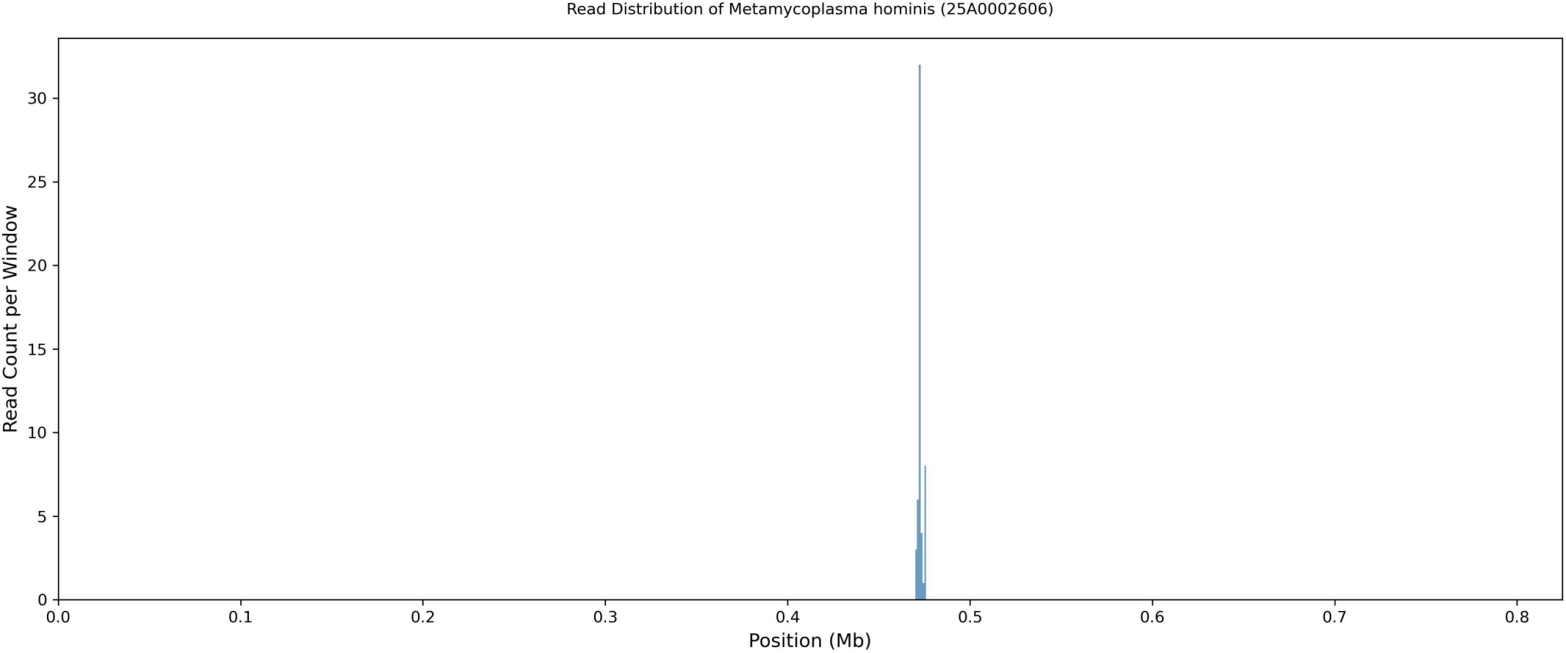
Read distribution of *M. hominis* detected by mNGS. The x-axis represents the genomic positions of the *M. hominis* reference genome (GenBank accession: GCA_009664325.1), and the y-axis indicates the number of mapped reads per 1,000 bp window. A total of 54 reads were mapped to the reference genome.

A total of 54 reads that mapped to *M. hominis* clustered within a narrow genomic region (**Figure 4**; **Supplementary Table 1**). Despite the low coverage, these reads were non-randomly clustered, exhibiting a distinct localized enrichment (hotspot) within a narrow genomic region of the reference genome (approximately at the 0.47 Mb position) (**Figure 4**; **Supplementary Figure 2**; **Supplementary Table 1**). No comparable clustering or enrichment of *M. hominis* reads was observed in the concurrently processed negative or extraction controls, supporting the analytical plausibility of this finding. Despite the low read abundance, this non-random clustering pattern was more consistent with a specific *M. hominis* signal than with diffuse background noise.

### 3.3 Interpretation of mNGS findings

mNGS was used to detect multiple organisms. Among the detected organisms, *M. hominis* and *U. parvum* were identified in both cases, whereas *P. bivia* was detected only in Case 2; all detected pathogens are known to be potential pathogens in postoperative gynecological infections (Chikamatsu et al., 2024; Kaya et al., 2024; Yagi et al., 2024). However, careful interpretation based on the antimicrobial response and clinical context allowed the exclusion of organisms other than *M. hominis* as the primary etiologic agent (Krausse and Schubert, 2010).

The clinical response to clindamycin was inconsistent with the involvement of *Ureaplasma* species, which are intrinsically resistant to this agent (Krausse and Schubert, 2010). In Case 2, the high read count of *P. bivia* was not interpreted as evidence of ongoing infection at the time of sampling because the specimen was collected during effective therapy for *P. bivia* chorioamnionitis and bacteremia. Accordingly, abundant *P. bivia* DNA was considered to be compatible with residual bacterial DNA rather than the persistence of infection. Therefore, within the same biphasic clinical course, *M. hominis* was regarded as the most plausible organism associated with the recurrent febrile phase and responded promptly to clindamycin.

In Case 1, the mNGS signal for *M. hominis* was quantitatively dominant and qualitatively reliable with unbiased genome-wide read mapping. The organism was also recovered by extended anaerobic culture, providing microbiological confirmation. However, only two pinpoint colonies grew, yielding barely sufficient material for MALDI-TOF identification and rendering antimicrobial susceptibility testing infeasible. Minimal colony growth would have likely been overlooked without the guidance provided by mNGS.

In Case 2, the interpretation was more challenging because the mNGS signal for *M. hominis* was extremely limited, with only a small number of reads detected. However, *M. hominis* was considered a plausible contributing organism based on both clinical findings and analytical features.

The detection of *M. hominis*, even at very low read counts, was concordant with the biphasic fever pattern, in which the initial fever improved with treatment for *P. bivia*, whereas the second febrile episode resolved promptly after the initiation of clindamycin. A previous report described a bloodstream infection diagnosed using as few as 19 reads (Chen et al., 2023), indicating that even low-abundance signals may have clinical significance when consistent with the clinical context.

The alignment pattern was non-random and exhibited a localized enrichment (hotspot) within a specific region of the reference genome (**Figure 4**; **Supplementary Figure 2**), exceeding the background levels observed in the controls. This pattern was not observed in the negative or extraction controls and was therefore distinct from background noise. Accordingly, this signal was considered analytically plausible and consistent with an organism-specific signal rather than nonspecific background.

The limited read count in this case may be explained, at least in part, by several factors. First, the specimen was expected to contain a high proportion of non-target DNA. The specimen was a small volume of purulent, blood-tinged intrauterine fluid (endometrial aspirate) obtained via a transcervical approach, which is expected to contain abundant host-derived DNA and background bacterial DNA, both of which may have reduced the relative detectability of low-abundance pathogens (Pereira-Marques et al., 2019). In addition, no host DNA depletion step, such as saponin treatment, was performed prior to sequencing, which may have further reduced the relative detectability of microbial reads. Second, the extracted DNA was stored under non-optimal conditions for a prolonged period. mNGS was performed retrospectively using extracted DNA that had been stored at 4 °C in RNase-free water for approximately one year. Low-concentration DNA is susceptible to degradation during storage, and prolonged storage in aqueous solution has been reported to result in loss of amplifiable DNA (Smith and Morin, 2005). Finally, *M. hominis* has a relatively small genome compared with most other bacteria, which may make it more susceptible to DNA degradation (Pereyre et al., 2009). Therefore, the low number of detected reads in this case may reflect not only low biological abundance but also storage-related degradation of DNA.

These findings indicate that *M. hominis* was the most plausible organism associated with both infections. The key differences between the two cases are summarized in **Supplementary Table 4**. The contrasting mNGS profiles of the two cases highlighted the importance of interpreting sequencing results within the clinical context and sample conditions.

## 4 Discussion

In this study, we describe two cases of postoperative pelvic infections associated with *M. hominis* that were identified using mNGS. These cases highlighted two key observations. First, the clinical relevance of organisms detected by mNGS cannot be judged by read numbers alone. Low-abundance reads may still represent clinically meaningful pathogens, whereas abundant reads may reflect residual DNA under effective therapy. Second, mNGS is particularly valuable in clinical contexts where conventional diagnostic methods often fail because of the presence of fastidious genital mollicutes and inherently mixed microbial DNA (Winand et al., 2019; Zhang et al., 2019; Nogami et al., 2023), such as in gynecologic pelvic infections. These observations contribute to the practical understanding of the interpretation and application of mNGS in clinical settings.

mNGS findings should be interpreted in conjunction with clinical and microbiological information, rather than solely based on absolute read counts. Recent studies have repeatedly emphasized that mNGS read counts are highly susceptible to several factors, including specimen sterility, host DNA proportion, antibiotic exposure, and storage conditions (Smith and Morin, 2005; Pereyre et al., 2009; Gu et al., 2019; Pereira-Marques et al., 2019) and thus, may be unreliable indicators of pathogenicity when interpreted without a clinical context (Simner et al., 2018). The two cases presented herein exemplify this principle. Both involved postoperative pelvic infections associated with *M. hominis*; however, the mNGS findings contrasted. In Case 1, abundant *M. hominis* reads with a genome-wide distribution were obtained, suggesting a reliable mNGS signal. In contrast, Case 2 yielded a very small number of *M. hominis* reads confined to a narrow genomic region. This discrepancy likely reflects differences in specimen characteristics and the pre-analytical conditions in Case 2, such as non-sterile transcervical sampling of blood-tinged, purulent, partially clotted intrauterine fluid (endometrial aspirate) and prolonged DNA storage (Smith and Morin, 2005; Pereyre et al., 2009; Pereira-Marques et al., 2019; Poulsen et al., 2021). Notably, the extracted DNA had been stored at 4 °C in RNase-free water for approximately one year prior to mNGS analysis, a condition that may affect nucleic acid stability. Previous studies have shown that prolonged storage at 4 °C is associated with reduced microbial read counts and decreased analytical sensitivity, particularly in low-microbial-biomass samples, potentially leading to underestimation of the true microbial burden (Diao et al., 2023). Although this represents a retrospective analysis under relatively extreme conditions, these findings underscore the importance of considering pre-analytical factors, including specimen storage conditions, when interpreting mNGS results. Both patients exhibited prompt improvement after empiric clindamycin treatment, which may have contributed to avoiding surgical intervention (Redelinghuys et al., 2014; Mori et al., 2016). Clindamycin was initiated before the mNGS results became available and therefore represents a limitation of this report. However, particularly in Case 2, ignoring the limited *M. hominis* signal could plausibly have resulted in a worse clinical outcome. This observation underscores that relying on read counts alone could lead to misleading etiologic interpretation and potentially inappropriate clinical decision-making.

mNGS is particularly valuable in clinical contexts where conventional diagnostic methods often fall short, with gynecological pelvic infections representing a typical example. This could be attributed to two major factors. The first is the frequent involvement of fastidious genital mollicutes such as *Mycoplasma* and *Ureaplasma* species in gynecological infections (Chikamatsu et al., 2024; Yagi et al., 2024). These organisms are notoriously difficult to detect using routine culture because they lack a cell wall, grow slowly, and form tiny colonies (Taylor-Robinson, 1996; Yeoh et al., 2023). The range of effective antimicrobial agents is limited; therefore, accurate species identification is essential for selecting an appropriate therapy (Krausse and Schubert, 2010; Valentine-King and Brown, 2017). Previous reports have documented severe and occasionally fatal outcomes associated with delayed diagnosis and ineffective treatment (García-de-la-Fuente et al., 2008; Reissier et al., 2016; Ali et al., 2021), underscoring the importance of recognizing these pathogens without delay. In addition, specimens collected in the setting of pelvic infections, including pelvic abscesses or endometrial tissue, often contain mixed microbial DNA (Smith and Morin, 2005; Moreno et al., 2018). In such samples, 16S rRNA PCR amplifies several templates simultaneously, resulting in overlapping chromatograms on Sanger sequencing, which preclude reliable sequence interpretation (Winand et al., 2019; Zhang et al., 2019). In both cases, 16S rRNA PCR yielded clear amplicon bands, whereas Sanger sequencing produced uninterpretable chromatograms. Because of these two factors, pelvic infections constitute a diagnostic “blind spot” in which both conventional culture and Sanger-based 16S rRNA sequencing may fail, particularly when genital mollicutes are involved. In this context, mNGS can successfully serve as a complementary diagnostic tool for identifying potential pathogens, as demonstrated in our two cases.

This study had several limitations that should be acknowledged. First, the limited number of cases from a single center inevitably limits the generalizability of our findings. Second, several methodological limitations should be acknowledged. In Case 2, mNGS was performed retrospectively using DNA that had been stored at 4 °C for one year, and degradation of bacterial DNA may have affected the quantitative read counts. In addition, no residual clinical specimen or extracted DNA remained after the original molecular analyses and mNGS workflow; therefore, we were unable to perform orthogonal confirmation such as *M. hominis*-specific qPCR/ddPCR, repeat testing, or serial quantitative assessment in Case 2. Third, the inference that mixed-background bacterial DNA interferes with 16S rRNA PCR/Sanger sequencing is based on indirect evidence, and the possibility of technical failure during the sequencing process cannot be entirely excluded. Fourth, mNGS findings did not directly influence therapeutic decisions. Therefore, this study had methodological limitations in terms of influencing the integration of mNGS into clinical decision-making processes. Finally, in interpreting the mNGS results, we considered organisms as causative agents primarily based on the following two considerations: (i) concordance with organisms known to cause pelvic infections and (ii) consistency with the observed clinical response to antibiotic therapy. No universally accepted standards for distinguishing true infections from colonization or contamination have been established (Gu et al., 2019; Nogami et al., 2023); thus, the interpretation of mNGS results necessarily relies on expert judgment, reflecting a limitation inherent to mNGS. However, this limitation reflects real-world clinical conditions and underscores the practical value of our observations for interpreting mNGS results in clinical settings.

In summary, the two postoperative pelvic infection cases described herein demonstrate that mNGS can serve as an adjunctive diagnostic tool in gynecologic pelvic infections, particularly when conventional methods fail to identify the causative pathogen. However, the clinical value of mNGS depends on careful interpretation of the results within the appropriate clinical context.

## Data Availability

The datasets presented in this study can be found in online repositories. The names of the repository/repositories and accession number(s) can be found below: https://www.ncbi.nlm.nih.gov/, PRJNA1429532; PRJNA1431772.
