# Supplementary material for: Diagnostic challenges in postoperative pelvic infections associated with Metamycoplasma hominis: a two-case analysis using metagenomic sequencing
Source: Front Cell Infect Microbiol. 2026 Apr 29;16:1823299. doi: 10.3389/fcimb.2026.1823299 (PMC13168059; doi:10.3389/fcimb.2026.1823299)
Supplement: Supplementary file 1 [file DataSheet1.pdf]

Supplementary Material

Table S1

Sequencing metrics of Patient's samples

Specimen_id	Through_put	host_ratio (%)	Microbe_reads	unclassified_reads
Case 1	82438021	99.85	126299	36602
Case 2	100,274,079	97.27	2,735,259	1,567,397

Table S2

Top10 microbial species in extraction control

Organism_name	reads	abundance (%)
Pelomonas aquatica	19510	16%
Burkholderia contaminans	9139	7%
Sphingomonas ursincola	9011	7%
Cutibacterium acnes	7440	6%
Faucicola osloensis	3666	3%
Geobacillus stearothermophilus	3454	3%
Thermoanaerobacterium thermosaccharolyticum	3309	3%
Staphylococcus epidermidis	2329	2%
Bradyrhizobium cosmicum	1974	2%
Escherichia coli	1875	1%

Table S3

Top10 microbial species in negative control

Organism_name	reads	abundance (%)
Cutibacterium acnes	161	9%
Sphingomonas ursincola	133	8%
Escherichia coli	46	3%
Acinetobacter junii	43	2%
Burkholderia cenocepacia	41	2%
Faucicola osloensis	41	2%
Nocardioides kribbensis	31	2%
Bosea massiliensis	29	2%
Caulobacter vibrioides	28	2%
Staphylococcus epidermidis	23	1%

Table S4

Structured comparison of the two cases, conventional microbiologic findings, key mNGS indicators, treatment, and outcomes

**Feature**	**Case1**	**Case2**
Clinical setting	Postoperative pelvic abscess following radical trachelectomy for cervical cancer	Postpartum endometritis following cesarean section, complicated by chorioamnionitis and *Prevotella bivia* bacteremia
Timing of specimen collection	POD 18	POD 6
Specimen	CT-guided pelvic abscess aspirate	Transcervical endometrial aspirate
Pre-analytical context for mNGS	mNGS performed during the acute clinical course	mNGS performed retrospectively using extracted DNA stored at 4 °C for approximately 1 year
Conventional culture	Initially negative at routine incubation; prolonged anaerobic culture later yielded two tiny colonies of *M. hominis*	Negative, including prolonged incubation
16S rRNA gene PCR/Sanger sequencing	PCR positive; Sanger sequencing uninterpretable because of mixed/overlapping chromatogram peaks	PCR positive; Sanger sequencing uninterpretable because of mixed/overlapping chromatogram peaks
Sequencing throughput (total reads)	82,438,021	100,274,079
Host-derived reads	99.85%	97.27%
Microbial reads after host subtraction	126,299	2,735,259
Target organism reads (*M. hominis*)	7,917 reads (~89% of bacterial reads)	54 reads
M. hominis genome coverage / mapping pattern	47% genome coverage with broad genome-wide distribution	0.18% genome coverage with localized clustering (hotspot)

Background / co-detected bacterial composition	Low-background profile; co-detected *Ureaplasma parvum* (905 reads) and *Lactobacillus iners* (5 reads)	Mixed bacterial profile dominated by *P. bivia* (163,983 reads), *U. parvum* (10,029 reads), and *Corynebacterium striatum* (559 reads)
Final treatment plan	Clindamycin monotherapy after empiric addition to piperacillin–tazobactam; total clindamycin duration 14 days	Meropenem continued for *P. bivia* bacteremia, with clindamycin added for endometritis; total durations of meropenem and clindamycin were 14 days each
Outcome	Marked improvement in fever and inflammatory markers after clindamycin initiation; no relapse during follow-up	Marked improvement in fever and abdominal pain after clindamycin addition; no relapse during follow-up

Figure S1

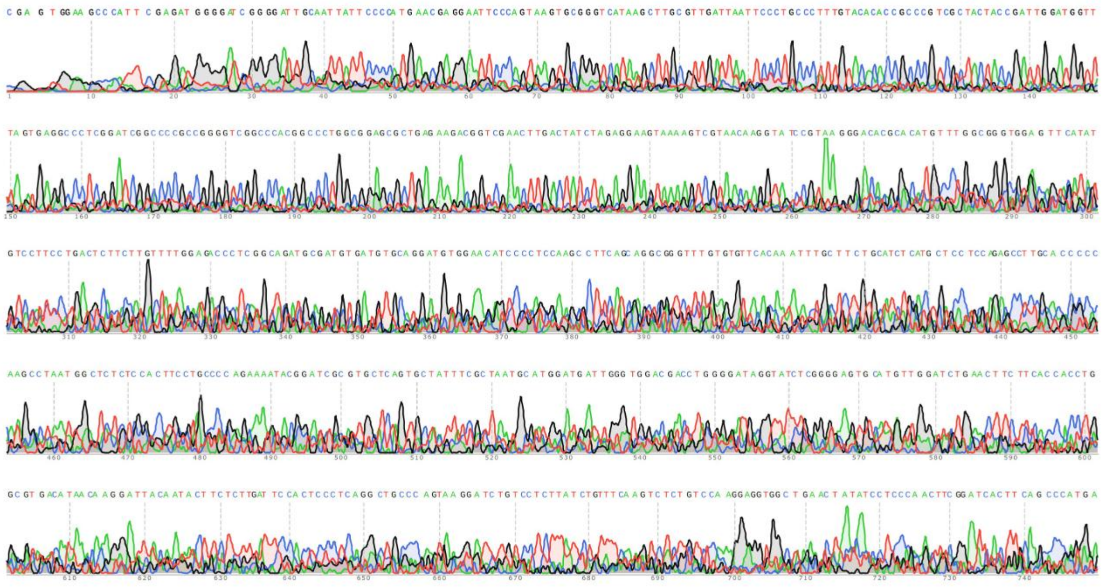

Figure S1. Uninterpretable Sanger sequencing chromatogram of the 16S rRNA gene.

Representative forward sequencing chromatogram obtained from the 16S rRNA gene PCR product. Multiple overlapping peaks were observed throughout the sequence, preventing reliable base calling and species identification. Similar uninterpretable results were obtained from reverse sequencing.

Figure S2a

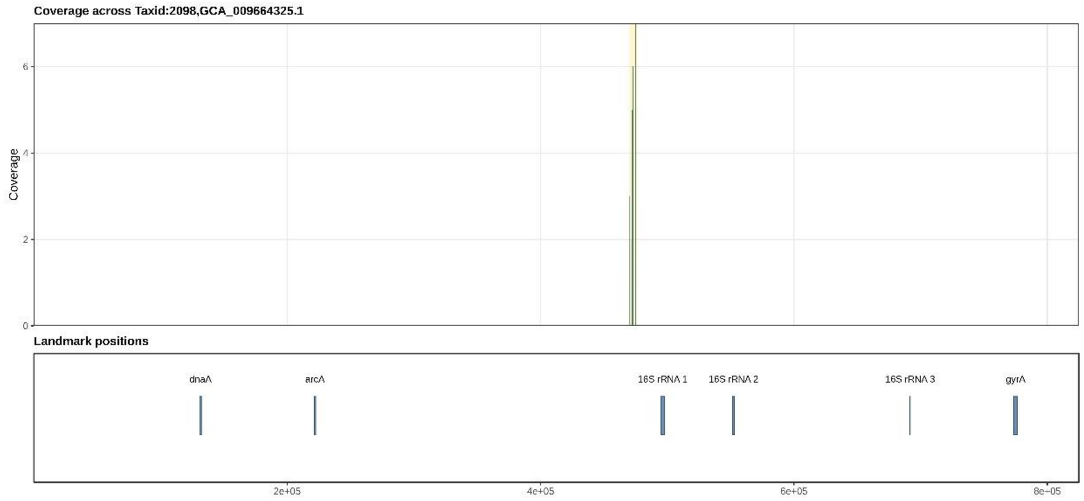

Figure S2b

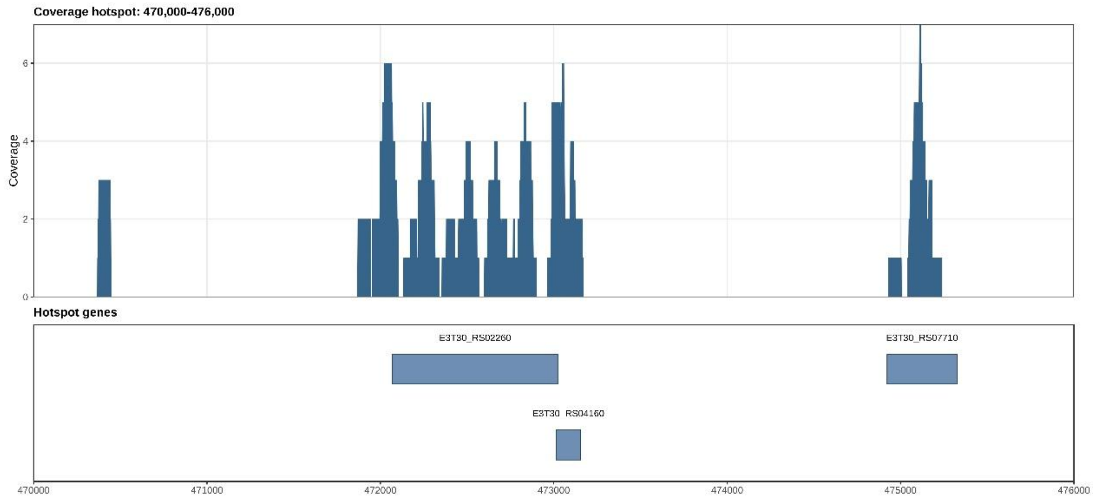

Figure S2. Genomic mapping and hotspot analysis of M. hominis reads in Case 2. **a,** Genome-wide read distribution. The x-axis represents the genomic coordinates of the *Metamycoplasma hominis* reference genome (GenBank: GCA_009664325.1), and the y-axis indicates the mapped read count per 1,000-bp bin. Although the overall abundance was low (54 total reads), the distribution was non-random, exhibiting a distinct localized enrichment. The positions of key marker genes, including *dnaA*, *arcA*, *16S rRNA* (1–3), and *gyrA*, are indicated. **b,** Zoomed-in view of the genomic hotspot (nt 470,000–476,000). This panel provides an expanded view of the primary mapping cluster (approximately at the 0.47 Mb position) to illustrate the high-density alignment. The schematic diagram below the x-axis identifies the specific genetic features (e.g., mobile genetic elements) associated with this hotspot.
