# Supplementary material for: Diagnostic challenges in postoperative pelvic infections associated with Metamycoplasma hominis: a two-case analysis using metagenomic sequencing
Source: Front Cell Infect Microbiol. 2026 Apr 29;16:1823299. doi: 10.3389/fcimb.2026.1823299 (PMC13168059; doi:10.3389/fcimb.2026.1823299)
Supplement: Supplementary file 2 [file DataSheet2.pdf]

# Supplementary Methods

## Metagenomic Sequencing and Analysis

### NUCLEIC ACID EXTRACTION

**Nucleic acid extraction from clinical Specimen**

Nucleic acids were extracted from clinical specimens using a commercially available nucleic acid extraction kit (MORA-EXTRACT; Advanced Microorganism Research Co., Ltd., Gifu, Japan), according to the manufacturer's instructions. This kit employs a combination of mechanical disruption using zirconia beads and phenol-based purification, enabling efficient recovery of nucleic acids from a broad range of microorganisms. The extracted DNA was eluted in RNase-free water. In both cases, DNA extraction was performed immediately after specimen collection. In Case 1, mNGS was performed as part of the diagnostic evaluation during the acute clinical course. In Case 2, the extracted DNA was subsequently stored at 4 °C for approximately one year prior to mNGS analysis.

### DNA LIBRARY CONSTRUCTION

**DNA Library Preparation**

Post-extraction, DNA concentration was quantified using the Qubit dsDNA HS Assay Kit on a Qubit 4.0 Fluorometer (Thermo Scientific, USA). For library construction, approximately 100 ng of genomic DNA was processed using the TIANSeq Direct Fast DNA Library Prep Kit (TIANGEN BIOTECH, Beijing, China) . The protocol involved enzymatic fragmentation and end-repair to yield an insert size distribution of 150–250 bp. Subsequently, Illumina-style UDI adapters, compatible with the Element AVITI sequencing platform, were ligated to the fragments. The libraries were amplified using a high-fidelity polymerase under the following thermal cycling conditions: initial denaturation at 95°C for 3 minutes; 12 cycles of 98°C for 20 s, 60°C for 15 s, and 72°C for 30 s; followed by a final extension at 72°C for 10 minutes. Post-amplification, the products were purified using 0.8× magnetic beads and eluted in TE buffer. Final library concentrations were verified via Qubit 4.0, with a threshold of ≥1 ng required for downstream sequencing.

### Library pooling and Sequencing

Qualified libraries were pooled based on equimolar concentrations targeting approximately **40 million reads per sample**. The pooled library was diluted according to the **AVITI 2x75 Sequencing Kit Cloudbreak FS High Output** specifications. Single-end sequencing (1×75 bp) was conducted according to the standard AVITI instrument protocol on the **Element Biosciences AVITI System**, using **Avidity Base Chemistry**, which provides high accuracy and low error rates.

## Bioinformatics Pipeline and Pathogen Identification

### Bioinformatic Processing and Quality Control

To ensure the integrity of the downstream analysis, raw high-throughput sequencing data were subjected to a rigorous quality-control protocol. We utilized Trimmomatic (v0.39) and PRINSEQ (v0.20.4) to exclude low-fidelity reads, specifically removing sequences shorter than 70 base pairs, those with a Phred quality score below 20 (Q20), PCR duplicates, and reads characterized by low sequence complexity. To eliminate host-derived background, the remaining high-quality reads were aligned to the Telomere-to-Telomere human reference genome (T2T-CHM13, v2.0) using the Burrows-Wheeler Aligner (BWA-MEM, v0.7.17) with default parameters.
Microbial Classification and Reference Database Reads that did not map to the human genome were extracted and subjected to secondary alignment against a specialized, non-redundant microbial database using BWA-MEM. This comprehensive reference library comprised 5,884 viral, 18,432 bacterial, 3,146 fungal, and 370 parasitic genomes. All genomic entries were meticulously curated from NCBI GenBank, RefSeq, and Virus databases, with a prioritization for high-quality, complete reference genomes to ensure taxonomic accuracy.

**Criteria for Pathogen Detection and Interpretation**

Candidates for potential pathogens were identified based on a series of stringent quantitative and qualitative filters:

- Normalized Abundance Threshold: For most taxa, a positive detection required a normalized abundance (Transcripts Per Million, TPM) at least 10-fold greater than the highest value observed in any concurrent negative controls, including extraction, library preparation, and no-template controls.
- Fastidious and Low-Yield Organisms: For specific pathogens known to be difficult to detect to low microbial biomass, slow growth, or challenges in nucleic acid extraction or sequencing (e.g., slow-growing bacteria such as *Mycobacterium tuberculosis*, certain RNA viruses, and low-biomass organisms such as genital mollicutes), a more sensitive threshold of at least one uniquely mapping read was applied, provided the taxon was entirely absent from all matched controls.
- Mapping Uniformity Assessment: To prevent false positives arising from repetitive elements or sequencing artifacts, we evaluated the spatial distribution of reads across the reference genome using Shannon entropy. When reads exhibited a relatively uniform distribution across the reference genome, corresponding to higher Shannon entropy values, the signal was considered reliable. In our dataset, such patterns were generally associated with higher entropy values (e.g., approximately >0.8). In contrast, when entropy values were lower and reads were concentrated within limited genomic regions, the signal was interpreted with caution. Such findings were not excluded solely based on entropy but were evaluated in conjunction with read counts, control comparisons, and clinical context.
